# Epigenetic Disregulation in Oral Cancer

**DOI:** 10.3390/ijms13022331

**Published:** 2012-02-21

**Authors:** Massimo Mascolo, Maria Siano, Gennaro Ilardi, Daniela Russo, Francesco Merolla, Gaetano De Rosa, Stefania Staibano

**Affiliations:** 1 Department of Biomorphological and Functional Sciences, Pathology Section, University of Naples “Federico II”, Naples 80131, Italy; E-Mails: mmascol@gmail.com (M.M.); dysian@tin.it (M.S.); gennaro.ilardi@unina.it (G.I.); danielarusso83@yahoo.it (D.R.); francesco.merolla@unina.it (F.M.); gaderosa@unina.it (G.D.R.); 2 Centro di Riferimento Oncologico di Basilicata (C.R.O.B.) Oncology Research Center of Basilicata, Rionero in Vulture, Potenza 85028, Italy

**Keywords:** epigenetics, oral cancer, tumor progression, prognosis, molecular therapy

## Abstract

Squamous cell carcinoma of the oral region (OSCC) is one of the most common and highly aggressive malignancies worldwide, despite the fact that significant results have been achieved during the last decades in its detection, prevention and treatment. Although many efforts have been made to define the molecular signatures that identify the clinical outcome of oral cancers, OSCC still lacks reliable prognostic molecular markers. Scientific evidence indicates that transition from normal epithelium to pre-malignancy, and finally to oral carcinoma, depends on the accumulation of genetic and epigenetic alterations in a multistep process. Unlike genetic alterations, epigenetic changes are heritable and potentially reversible. The most common examples of such changes are DNA methylation, histone modification, and small non-coding RNAs. Although several epigenetic changes have been currently linked to OSCC initiation and progression, they have been only partially characterized. Over the last decade, it has been demonstrated that especially aberrant DNA methylation plays a critical role in oral cancer. The major goal of the present paper is to review the recent literature about the epigenetic modifications contribution in early and later phases of OSCC malignant transformation; in particular we point out the current evidence of epigenetic marks as novel markers for early diagnosis and prognosis as well as potential therapeutic targets in oral cancer.

## 1. Introduction

Head and neck cancers constitute the sixth most common malignant tumors worldwide, affecting approximately 650,000 people and causing almost 350,000 cancer deaths per year [1,2]. These malignancies encompass tumors arising from the epithelium of the nasal and oral cavity, paranasal sinus, pharynx and larynx. Oral cancer is the most frequent cancer of the head and neck district, with squamous cell carcinoma being by far the commonest single entity, accounting alone for about 90% of all malignancies of the oral cavity [3]. Due to its related high mortality and low cure rate, oral squamous cell carcinoma (OSCC) represents a major public health problem, with a great individual and socioeconomic impact. In fact, despite of the many advancements made in the field of oral cancer prevention and multimodality treatments, the five-year survival rate for OSCC remains at a disappointingly stable level, almost unchanged over the past 20 years [4,5]. The poor prognosis of OSCC is mainly due to a low response rate to current therapeutic strategies, particularly for tumors diagnosed in advanced stage. This specific subset of patients is characterized by a high occurrence of invasion to surrounding tissues, lymph node and distant metastasis and by a peculiarly high risk of second malignancy during the patient’s lifetime.

Oral carcinogenesis is a multistep process modulated by endogenous and environmental factors. Among these latter, a major role is played by tobacco and alcohol regular intake [6], as well as by Human Papillomavirus (HPV) persistent infection [7–11]. These predisposing factors may lead to a wide range of genetic and epigenetic events that promote genomic instability and tumor development and progression. The genetic alterations involved in the development and progression of oral premalignancy and OSCC, are caused by irreversible changes in DNA sequence including gene deletions, amplifications and mutations leading both to oncogenes activation or tumor suppressor genes inactivation [12,13].

Epigenetics is another major player in multistep carcinogenesis of oral cancers. Here we discuss the current literature in the field of the epigenetics of oral cancer, placing a great deal of emphasis on DNA methylation, histone modification and post-transcriptional gene down-regulation by microRNAs. The emerging but still debated role of high-risk HPV persistent infections in determining a different subset of OSCC will also be discussed, reviewing some of recent publications about this topic and pointing out the relationship between HPV infection and cancer prone epigenetic modifications. We finally aim to highlight the important translational implications of the epigenetic regulation as new diagnostic, prognostic and predictive markers in oral cancer, an actually growing field of research due to the poor reliability of conventional markers in OSCC diagnosis, treatment and follow-up [12].

## 2. Epigenetics

The epigenetic changes refer to any heritable modifications in gene expression without alterations of the DNA sequence; they occur more frequently than gene mutations and may persist for the entire cell life and even for multiple generations [14]. The transcription of each gene may change from high-level expression to complete silencing, depending on the influence of the “epimutations” which interfere with the action of activators and suppressors on specific promoters in the chromatin context [15]. With minor exceptions (T- and B-cells of the immune system), all differentiation processes are triggered and maintained through epigenetic mechanisms. Epigenetic inheritance includes DNA methylation, histone modifications and RNA-mediated silencing. Disruption of any of these three distinct and mutually reinforcing epigenetic mechanisms leads to inappropriate gene expression, resulting in cancer development and other “epigenetic diseases” [16–19] (Table 1).

## 3. DNA Methylation

DNA methylation is the most common and the best-studied epigenetic modification [20]. It is mediated by different DNA methyltransferases (DNMT) [21] and usually involves lysine and arginine residues on histone tails. The methylation of DNA refers to the covalent addition of a methyl group to the 5-carbon (C5) position of cytosine bases that are located 5′ to a guanosine base in a CpG dinucleotide. CpG dinucleotides, scattered throughout the genome, are usually found clustered in 0.5–4 kb regions, named CpG islands, the major part of which localized at the promoter of tumor suppressor genes [22]. CpG islands of growth-regulatory genes promoter regions are often found hypermethylated in tumors, this event causing the transcriptional “silencing” of tumor suppressor genes [23,24], contributing to cancer progression; on the contrary, it has also been described the derepression of proto-oncogenes transcription by hypo/demethylation, this leading to increased mutation rates and to chromosome instability, which constitutes an early hallmark of tumor cells [25–27]. Moreover, the loss of function of tumor-suppressor genes, which often occurs in tumors, has been ascribed more frequently to epigenetic silencing through methylation than to genetic defects [20,28], supporting the hypothesis that epigenetic alterations have a significant role in every step of carcinogenesis. The genes most frequently hypermethylated and silenced in cancer cells reside in chromosome regions commonly showing loss of heterozygosity. The LOH of hypermethylated genes may provide a selective growth advantage to tumor cells, and is often involved in metastatic ability and in tumor neo-angiogenesis [29].

## 4. DNA Methylation in Oral Carcinogenesis

Although a clear correlation between the epigenetic-driven deregulation of gene expression and the oral cancer progression is at present not fully demonstrated, hypermethylation and consequent silencing of several tumor suppressor genes, out of a group of more than 40 genes, has been identified in OSCC [20] (Table 2); the genes found hypermethylated in OSCC cover a wide range of cellular processes, including cell cycle control (p16, p15), apoptosis (p14, DAPK, p73 and RASSF1A), Wnt signalling (APC, WIF1, RUNX3), cell-cell adhesion (E-cadherin), and DNA-repair (MGMT and hMLH1) [30–33].

### 4.1. CDKN2A

The methylation rate of CDKN2A has been widely investigated and reported in the literature. CDKN2A gene maps on chromosome 9p21 and encodes the cell cycle regulatory protein p16, which inhibits the cyclin-dependent kinase 4 and 6 activity, inducing cell-cycle arrest in the G1 phase. The reported incidence of hypermethylation of p16 ranges from 23% to 76% in OSCC [41,43,54–58]. Some studies examined also this phenomenon in oral mucosa with different degree of dysplasia (pre-neoplastic lesions, OIN) [59,60] and in normal adjacent mucosa, showing higher values of hypermethylation in OIN compared to normal mucosa, but lower than in the OSCC.

### 4.2. E-Cadherin and N-Cadherin

CDH1 gene (cadherin 1 type 1) is located on chromosome 16q22.1 and encodes for E-cadherin, a 120-kd single-span transmembrane glycoprotein, with five extracellular and one cytoplasmic domain, interacting with catenins. This molecule is mainly involved in the formation of adhesive junctions in epithelial cells, playing a fundamental role in in many aspects of the establishment and maintenance of intercellular adhesion, cell polarity, intracellular signaling and tissue architecture. Several studies evaluating E-cadherin expression in different carcinomas, documented the crucial role of this molecule during tumor progression and invasion. In fact E-cadherin absence is strictly linked to alterations in cell key functions and motility. In addition it is shown that the loss of its expression is frequently involved in tissue metastasis. Similarly to others malignancies, it was shown a correlation between low expression of E-cadherin and a more aggressive behaviour of OSCC. Hypermethylation of CDH1 was also extensively reported [41,57,61–63]: in these studies the E-cadherin gene hypermethylation frequency ranged between 7% and 46% [30,64–65]. In a recent review, Vered *et al*. analyzed the recent literature evaluating the immunoexpression of E-cadherin in OSCC, highlighting the confusion existing about the expression of this protein in normal and neoplastic tissue. They stressed the need for a critical review of the IHC-based expression evaluation of this molecule, in order to better define the association between its expression and clinical outcome [66].

In a recent study, Di Domenico M examined N-cadherin expression, a calcium-dependent adhesion protein, in series of 94 OSCC. Neoplastic tissue showed a significantly higher expression of this protein, almost exclusively cytoplasmic, than normal tissue. In addition tumors with high N-cadherin value were characterized by a more aggressive behaviour. These data suggested that N-cadherin could have a potential role in predicting the biological behavior of SCC [67].

### 4.3. PTEN

PTEN (phosphatase and tensin homolog deleted on chromosome 10) is a tumor-suppressor gene located on chromosome 10q23.3, the loss of which expression is thought to be involved in important cellular processes including survival, differentiation, proliferation, apoptosis and invasion. In addition, due to lack of control of the signaling pathways that mediate apoptosis and migration, such as Ras/phosphoinositide 3-kinase (PI3K)/AKT, it plays a fundamental role in tumor cell survival and proliferation and metastasis. PTEN is frequently deficient in several malignancies because of mutations or epigenetic changes [68,69] In addition evidences has also been provided supporting that CpG islands of the *PTEN* promoter are methylated in several type of human cancers, such as endometrial carcinoma [70], gastric [71], non-small-cell lung carcinoma [72] and cervical cancer [73]. Kurasawa *et al*. analyzed the immunohistochemical expression of PTEN in 113 OSCC and 9 OSCC cell-lines [47]. The resulting data showed a significant difference of expression between tumor samples and normal tissues. No mutations were showed, but in 4 out 6 OSCC cell lines a lower expression of PTEN mRNA were observed. Taken together these results supported the hypothesis that PTEN plays an important role in OSCC pathogenesis and that its down-regulation is due to hypermethylation [47]. However, the role of PTEN in OSCC remains uncertain. Squarize *et al*. demonstrated that aggressive OSCC did not express PTEN [48] and Shin *et al*. showed that the genetic or epigenetic inactivation of PTEN was related to OSCC carcinogenesis [49], while several authors didn’t support this association [50,74,75]. Several studies have investigated the role of pTEN in OSCC and correlated the abnormal expression of PTEN to the occurrence, development and invasion of OSCC [76]. In a recent review, Diez-Perez *et al*. report the data relative to a comparison study between oral cancer tissue and normal mucosa, showing a 77.8% reduction of gene expression, due to its promoter methylation [42].

### 4.4. P53

TP53 gene maps on chromosome 17p13.1 and encode a tumor suppressor protein, also called p53, involves in many fundamental cell processes, such as cell cycle progression, cellular differentiation, DNA-repair and apoptosis. When an endogenous or exogenous stress occurs, p53 levels increase and lead to block cell cycle, allowing the DNA-repair. Loss of p53 function alters the ability of cells to respond to stress or damage, leading to genomic instability. P53 is mutated in the majority of human cancers, including oral cancers, with frequency ranging from 25% to 69% [77–80]. In several instances, however, p53 shows a loss of function due to epigenetic events, instead of genetic alterations. This is the case of the epigenetic inactivation of p53 non mutated protein by the E6 protein of high-risk HPVs (mostly HPV16 and 18), in OSCC as in some laryngeal cancers (see below for further discussion about this topic).

### 4.5. DAPK1

DAPK1 (death associated protein kinase 1) gene maps on chromosome 9q34.1, it encodes a pro-apoptotic calcium/calmodulin regulated serine/threonine kinase inducing apoptosis (p53-dependent apoptotic checkpoint) [43,58,81]. The reported frequency of DAPK promoter hypermethylation ranges from 18% to 27% [64,82,83].

### 4.6. MGMT

MGMT gene is located on chromosome 10q26, it encodes MGMT (06-methylguanine-DNA methyl transferase), a DNA repair enzyme that removes adducts caused by alkylating agents; such DNA repair activity favors the resistance of cells to treatment-induced apoptosis. Silencing this gene allows alkylated guanine to accumulate, restoring apoptosis [43,58]. The frequency of hypermethylation in OSCC ranges from 7% to 68% [41,43,58,65,82–85].

### 4.7. RARB2

RARB2 (retinoic acid receptor B2 gene) is a tumor suppressor gene belonging to the RARB family, mapping on chromosome 3p24. It is frequently inactivated in cancer, mainly by methylation. It is linked with the deregulation of cell proliferation in tumors as well as in preneoplastic lesions. Promoter methylation of the *RARB2* gene was generally reported in around 55% of lung SCLC, in 19% of urothelial carcinoma, in 27.5% of breast carcinomas, in prostate cancers, in endometrial carcinomas, colorectal carcinomas and it has been significantly associated with aggressive tumor phenotypes and patients survival in salivary glands carcinomas. RARB2 promoter methylation has been observed also in cancers of the head and neck region (67%) and in a significant percentage of precancerous lesions of the same district (>50%) [86]. Very interestingly, recent reports indicate that RARB2 methylation is independent from tumor site or stage, but is related to the higher age of patients (probably as an expression of the long time of action of carcinogens on the mucosal epithelium) and to the aggressiveness of tumors and poor prognosis of patients [45].

### 4.8. RASSF1 and RASSF2

RASSF1 (3p21.3) and RASSF2 (20p13) belong to the Ras association family (RASSF) of proteins involved in the Ras/PI3K/AKT pathways. Huang *et al*. showed that in almost the 50% of patients treated with radiotherapy Ras/PI3K/AKT pathways were activated in association with RASSF1A/RASSF2A gene silencing through promoter methylation [51]. In addition, Imai T *et al*. found RASSF2 methylated in 26% of OSCC evaluated [87].

Hypermethylation of p14ARK, p16^INK4a^, p15, MGMT, DAPK, GSTP1, RARB and p53 have been found in dysplasia and in histologically normal appearing margin of OSCC resection. Several studies suggest that methylation could be considered as an early promising marker of malignant progression. However, other studies have failed to correlate the hypermethylated state with progression toward cancer of OINs or to recurrence of OSCC in the site of excision. Therefore, this aspect deserves further investigation on a greater series of cases [12,43,58,59].

Less is known on the presence and role of hypomethylation: to date, it has been reported only the possible occurrence of SFRP1 (secreted frizzled-related protein 1) (8p11.21) hypometylation in OSCC, but the data are too few to achieve an overview of the phenomenon in these tumors. Contrary to Sogabe *et al*. who observed that SFRP1 together with SFRP2 and SFRP5 were methylated in OSCC, Pannone *et al*. found SFRP1 significantly demethylated in cancer (*p* < 0.05) [52].

## 5. miRNA

MicroRNAs (miRNAs) are a recently discovered class of non-coding endogenous small RNAs [88,89] which have a crucial role in the control of gene expression and are associated with promotion and progression of malignancies [90]. They are involved in many fundamental cellular processes such as proliferation, development, differentiation and apopotosis in normal and neoplastic cells, where they are referred to as oncomiRs (oncogenic miRNA). They act as mediators of epigenetic gene regulation, by interacting with mRNA, either by inhibiting mRNA translation or causing mRNA degradation [91]. Recent studies have been shown that miRNAs act as putative tumor suppressors and may also undergo epigenetic silencing in cancer [92,93]. Although there are still few studies focusing on the miRNA involvement in oral carcinogenesis, the interest about their functional roles in OSCC is rapidly growing. Cervigne *et al*. examined microRNA (miR) expression changes in 43 sequential progressive oral leukoplakia samples from 12 patients and 4 non-progressive leukoplakias from 4 different patients [94]. The findings were validated using quantitative RT-PCR in an independent cohort of 52 progressive dysplasias and oral squamous cell carcinomas (OSCCs), and 5 non-progressive dysplasias [94]. Global miR expression profiles distinguished progressive leukoplakia/OSCC from non-progressive leukoplakias/normal tissues. Of 109 miRs, which were highly expressed exclusively in progressive leukoplakia and invasive OSCC, miR21, miR181b, and miR345 expression was consistently increased and associated with increases in lesion severity during progression. The authors hypothesized that overexpression of miR21, miR181b, and miR345 may play an important role in malignant transformation [94]. Wong TS found that the level of miR-133a and miR-133B was significantly decreased in OSCC when compared with normal epithelia samples. These low levels led to the activation of a potential oncogene piruvate kinase type M2 [95]. Kozaki *et al*. investigated the miR-137 and miR-193a expression levels alteration in some OSCC cell lines, demonstrating that the epigenetic silencing of both miRNA, caused by DNA hypermethylation, could have a key function in oral cancer progression [46]. miR-17-92 was shown to play pleiotropic functions during both normal development and malignant transformation; it was demonstrated that the expression levels of mir-17-92 polycistronic cluster increased in cultured carcinoma cell lines in comparison to normal human keratinocytes [96,97]. Hu *et al*. demonstrated that miR-504 plays an important role during carcinogenesis, acting as a negative regulator of p53. In fact overexpression of miR-504 causes low demonstrated that miR-504 plays an important role during levels of the tumor suppressor [98]. The potential role of miR-504 as new diagnostic, prognostic and therapeutic tools has been recently discussed by Wu *et al*. and by Gorennchtein *et al*., both hypothesizing a clinic advantage in OSCC patient management [99,100]. Table 3 summarizes several miRNAs whose expression is deregulated in OSCC. The accumulating data about this topic let envisage mi-RNAs as one of the most valuable biomarkers and therapeutic targets in current OSCC research. During recent years, the trend of research in mi-RNA and OSCC field has evolved from solely searching altered specific miRNAs to exploring molecular networks and connections between miRNAs and signaling pathways involved in the progression of OSCC.

## 6. Chromatin Dynamics and Histones Modifications

**Figure 1:**
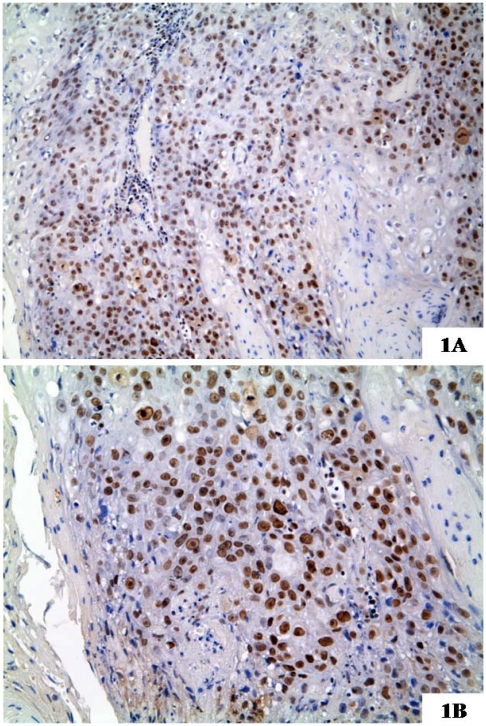
A case of aggressive squamous cell carcinoma of tongue, Human Papillomavirus (HPV)-negative, showing a strong expression of chromatin Assembly Factor-1 (CAF-1)/p60. The patient experienced two local recurrences, nodal and distant metastases and died for disease in a period between 1 and 3 years from diagnosis. In the higher magnification you can note most nuclei of tumor cells strongly immunostained for the protein (1A: original magnification × 200; 1B: original magnification × 250).

The chromatin structure is highly regulated by complex interactions between many molecular pathways, that influence normal and tumor cell fate, for that concerning DNA replication, transcription, and repair, cell growth and differentiation, apoptosis and every crucial cell functions. Histones and chromatin modifiers mainly induce changes of chromatin architecture. Histones have a structural role in the chromatin architecture entering into the constitution of nucleosomes. Acetylation, methylation, phosphorylation and ubiquitination are major histone modifications, combination of which may constitute the “histone code” that extends and modulates the genetic code [101,102]. Among molecules that regulate the chromatin assembly, histone chaperones play an essential role. They drive histones incorporation into newly synthesized or remodeled chromatin [103]. In this process, the Chromatin Assembly Factor-1 (CAF-1) exerts a pivotal role: it destabilizes heterochromatic structures during replication, allowing the replication machinery to progress through heterochromatin. CAF-1 is a protein complex, formed of three subunits with different molecular weight (p48, p60 and p150) and delivers histones H3 and H4 on newly synthesized DNA [104] during DNA replication and DNA repair. CAF-1 facilitates the incorporation and assembly into chromatin of H3K56 acetylated histones, in response to oxidative stress [105] and DNA damage [106–108]; moreover it contributes to resolve the mismatch-containing strands, restoring chromatin structure on the completion of double strand break repair [109]. In particular, the p48 subunit works as partner for the retinoblastoma protein RbAp48, participating in cell-growth suppression, and is involved in histone metabolism [110]; the p150 and p60 subunits are essential for the S-phase progression [111,112] and for the restoration of the original chromatin organization following DNA repair [113]. Moreover, p150 protein directly interacts with the proliferating cell nuclear antigen (PCNA) and is actively involved in proliferation-linked DNA repair processes [114]; the p60 subunit is highly required for the efficient progression through S-phase and, as expected, it is down-regulated in quiescent cells, and overexpressed in neoplastic cells [103]. CAF1/p60 has been found overexpressed in a series of human malignancies, including breast, prostate, melanoma, salivary glands, cervical, endometrial and renal cell cancers, in close association with their biological aggressiveness [111,115–119]. The role of CAF-1/p60 as new prognostic marker in oral tongue SCC has been also investigated. CAF1/p60 expression has been found significantly higher in OSCC with a worse prognosis, allowing the authors to propone this protein as a useful tool for evaluation of biological behavior of these tumors [118] [Figure 1].

Several lines of evidence implicated histone acetylation in human malignancies. The histone acetylation status mainly depends on the activity of histone acetyltransferases (HATs) and histone deacetylases (HDACs). Some recent works evaluated the role of histone deacetylase (HDAC) inhibitors in OSCC cell lines and OSCC tissues [120–127]. HDAC inhibitors are a new group of anti-neoplastic drugs that promote acetylation of core histones, leading in turn to the uncoiling of chromatin and activation of several genes involved in the regulation mechanisms of cell survival, proliferation, differentiation, and apoptosis. Sakuma *et al*. found a higher expression of deacetylase 6 in OSCC cell lines and OSCC tissue samples in comparison to normal oral tissue and nine OSCC cell lines [120]. Moreover, Sato *et al*. highlighted the importance of timing of addition of HDAC inhibitors when used in combination with cisplatin, in a study performed on OSCC cell lines. In fact, CDDP-treated cells displayed varying degrees of apoptotic responses depending on timing of HDAC inhibitors addition [121]. Rihiishi *et al*. observed a cooperative effect between histone deacetylase (HDAC) inhibitor (suberoylanilide hydroxamic acid) on cisplatin (CDDP)-induced apoptosis on human OSCC cell lines [122]. Chang *et al*. evaluated the expression of Histone deacetylase 2 protein on 20 cases of oral epithelial dysplasia and 93 cases of OSCC [123]. In consideration of the resulting data showing that overexpression of the HDAC protein is a frequent event in OSCC, they suggested that Histone deacetylase 2 protein could be used as a prognostic factor in oral SCC. Based on reports supporting the useful association between HDAC inhibitors and some traditional chemotherapeutic agents, Shen and colleagues evaluated the potential combinative effect of low dose cisplatin andsuberoylanilide hydroxamic acid, in OSCC cell lines [125]. The authors concluded that concurrent treatment with SAHA enhanced tumor cell sensitivity to subtoxic doses of cisplatin.

## 7. Human Papilloma Virus (HPV)

High Risk Papillomavirus (HR-HPV) persistent infection has been recently indicated as an independent risk factor for head and neck cancers and, although it is still a “hot” debated topic, a growing body of literature has documented the link between high risk papillomavirus (HR-HPV) persistent infection of oral epithelium and the development of OSCC [7,8,128–132]. Miller and Johnstone, the first to publish a meta-analysis on HPV prevalence in precancer lesions, cancer and normal oral mucosa, showed that HPV was 2–3 times more likely to be detected in oral precancer lesions, and 4.7 times more likely to be present in oral carcinomas, when compared with normal mucosa [133]. Syrjänen reviewed the HPV literature published prior to 1998, and the pooled HPV detection rates in normal oral mucosa, OL and OSCC ranged from 13% to 33% depending on the technique used to detect HPV (PCR, ISH or both) [134].

In a recent meta-analysis study, Syrjänen *et al*. showed that HPV significantly increases the risk for OSCC, as compared with the controls (OR 3.98, 95% CI: 2.62–6.02); nevertheless, in the total lack of prospective cohort studies, the authors were unable to take a position on the temporal relationship between HPV infection and oral malignancies [135]. The results of this meta-analysis showed a strong association between HPV and OSCC in accordance with previous studies [131–138].

The association between high risk papillomavirus (HR-HPV) persistent infection of oral epithelium and the development of OSCC characterizes a distinct subgroup of malignancy arisen in a younger and higher socio-economic group, often non-smokers/non-drinkers [139]. This subset of HR-HPV positive lesions shows different genetic and molecular profile and has been associated with a more favorable prognosis, compared to the HPV-negative ones, although more often they are poorly differentiated. In fact, HR-HPV-associated OSCCs are characterized by a significantly lower malignant progression rate and by a better responsiveness to chemo-and radiotherapy when compared with tumors of the same grade and stage, negative for viral infection [140,141]. However, it is not yet well known if the better biological behavior and the better response to the adjuvant therapies of the HR-HPV-associated OSCC might be due to the indirect action of innate or acquired cofactors, moreover to formally confirm the role of HPV as an etiological agent of OSCC, additional evidence is required.

From the molecular point of view, HR-HPV+ tumors are characterized by high genomic instability, due to a dysregulation of cell cycle control, epigenetically induced by the E6 and E7 HR-HPV oncoproteins [142]. E6 and E7 proteins bind and degrade, respectively, p53 and the retinoblastoma (pRb) proteins, leading to the continuous expression of the cyclin-dependent kinases CDK4 and CDK6 [143], bypassing then the normal checkpoints. In particular, the degradation of pRb causes, through a positive feedback mechanism, the increase of intracellular protein p16INK4a and leads to deregulated tumor cell proliferation [144,145]. These alterations have a dramatical impact on the oral mucosa, driving its malignant transformation. Nevertheless, it is very intriguing to note that they involve the same genes commonly mutated in OSCC due to the classical risk factors (alcohol and tobacco use). Several host epigenetic changes have been ascribed to HPV virus. The HPV appears to increase the *de novo* methyltransferase, DNMT3b protein; although no HPV protein has been identified as the culprit for this increase in DNMT3b, a recent study has now shown that HPV E7-protein increases DNA methyltransferase enzymatic activity by directly interacting with DNMT1. E7 protein has also been described to bind HDACs and Nurd ATP-dependent remodeling complex. Moreover, E6 protein binds and inhibits p300/CBP HAT [146–149].

The existence of a different clinical course among OSCCs arisen in HR-HPV+ and HPV− patients, confirm the hypothesis that epimutations and genetic mutations may have different consequences on the biological behavior of tumors, even if they tend to occur in a combined manner in most of human malignancies.

## 8. Considerations

Unlike genetic alterations, epigenetic changes are potentially reversible, and this feature makes them attractive targets for therapeutic intervention. Recent progresses in the knowledge of epigenetics of cancer have allowed the development of several inhibitors of DNA methiltransferase (DNMT), such as 5-azacitidine and decitabine, and histone deacetylase, successfully used in the treatment of several malignancies of the hematopoietic system, lung and even HNC [150–153]. The US NIH repository for clinical trials reports some trials involving epigenetic-based drugs in head and neck cancer treatment: Azacitidine and Cisplatin have been tested in combined chemotherapy in advanced, recurrent and metastatic squamous cell carcinoma of head and neck but no data are available to date about the outcome of the study [154]. DNA methylation has been demonstrated to concur in the silencing of chemoresistance related genes; demethylating agents could improve the sensitivity of cancer cells to anticancer drugs [155]. The use of epigenetic inhibitors in association with traditional anticancer therapeutic agents looks very promising as a tool to improve the chemosensitivity of non-responsive cancers [156]. A peculiar clinical benefit in OSCC, might be expected, for example, from the synergistic effect of epigenetic silencing of RASSF1 and radiotherapy to minimize radioresistance in OSCC [51]. Unfortunately, to date most of these therapeutic agents have shown some drawbacks, being toxic *in vitro* and *in vivo*. Nevertheless, the chance to counteract epigenetically-driven alterations in cancer cells opens an exciting scenario for its possible future fall-out on OSCC patients’ care.

## Figures and Tables

**Table 1:** The most common epigenetic alterations.

Epigenetic change	Putative mechanism	Biological consequence
DNA hypomethylation	Activation of cellular oncogenes	Increased proliferation, growth advantage
	Activation of transposable element	Genomic instability, transcriptional noise
DNA hypermethylation	De novo hypermethylation of CpG islands within gene promoters leading to silencing of tumor suppressors and cancer-associated genes	Genomic and chromosomal instability, increased proliferation, growth advantage
Loss of imprinting (LOI)	Reactivation of silent alleles, biallelic expression of imprinted genes	Expansion of precursor cell population
Relaxation of X-chromosome inactivation	Mechanisms is unknown but it appears to be age-related	Altered gene dosage, growth advantage
Histone acetylation	Gain-of-function	Activation of tumor promoting genes
	Loss-of-function	Defects in DNA repair and checkpoints
Histone deacetylation	Silencing of tumor suppressor genes	Genomic instability, increased proliferation
Histone methylation	Loss of heritable patterns of gene expression (“cellular memory”)	Genomic instability, growth advantage
MicroRNAs (miRNAs) amplification in cancer	Function as oncogenes	Neoplastic transformation
MicroRNAs (miRNAs) deletion in cancer	Function as tumor suppressors.	Neoplastic transformation

**Table 2:** The most common genes silenced from promoter methylation.

Gene	Locus	Function	Alterations	Ref
***ABO***	9q34	Blood group antigen	Hypermethilation	34
***APC***	5q21	Signal transduction	Hypermethilation	33,35
***ATM***	11q22-q23	Tumor suppressor	Hypermethilation	4,30,36,37
***C/EBPα***	19q13	Tumor suppressor	Hypermethilation	38
***CDKN2A***	9p21	Cell cycle	LOH, hypermethilation	4,39
***CRABP2***	1q21	Nuclear transcriptional regulator	Hypermethilation	40
***DAPK***	9q	Apoptosis	Hypermethilation	4,30,32,34,35,37
***DCC***	18q21	Tumor suppressor	Hypermethilation	4,30,34,37
***DKK3***	11p	Transcriptional regulator	Hypermethilation	12
***E-cadherin***	16q22	Signal transduction	Hypermethilation	4,32,41,42,34,36
***EDNRB***	13q22	Signal transduction	Hypermethilation	39
***GSTP1***	11q13	Detoxification of carcinogens	Hypermethilation	30,43
***H3K4***	1q21.2	Histone	Hypermethilation	44
***HIN1***	12p13	Tumor suppressor	Hypermethilation	45
***Hmlh1***	3p21	DNA repair	Hypermethilation	30,32,34,36
***LHX6***	9q33	Transcriptional regulator	Hypermethilation	38
***MGMT***	10q26	DNA repair	Hypermethilation	4,30,32,41,35,37
***MINT family***	/	/	Hypermethilation	30
***miR137***	1p21.3	Tumor suppressor	Hypermethilation	46
***miR193a***	17q11.2	Tumor suppressor	Hypermethilation	46
***MX1***	21q22	/	Hypermethilation	40
***p14***	9p21	Apoptosis	LOH, hypermethilation	30,34
***p15***	9p21	Cell cycle	LOH, deletion, mutation, hypermethilation	4,37
***p16***	9p21	Cell cycle	LOH, mutation, deletion, hypermethilation	30,32,35,38
***p53***	17p13	Tumor suppressor	Mutation, hypermethilation	30
***p73***	1p36	Apoptosis	Hypermethilation	30,34,35,38
***PTEN***	10q23	Tumor suppressor	Hypermethilation	47,50
***RARB2***	17q21	Nuclear transcriptional Regulator	Hypermethilation	4,30,42,34,35,37
***RASSF-1***	3p21	Apoptosis	Hypermethilation	4,32,51,37
***Rb***	13q14	Tumor suppressor	Hypermethilation, mutation	35
***RUNX3***	1p36	Transcriptional regulator	Hypermethilation	32,39
***SFRP1***	8p11.21	Transcriptional regulator	Hypometilation	52
***SFRP1-2-4-5***	8p11.21 4q31.3 7p14.1 10q24.1	Transcriptional regulator	Hypermethilation	52,38,53
***TCF21***	6q23-q24	epithelial-mesenchymal interactions	Hypermethilation	38
***THBS1***	15q15	cell-to-cell and cell-to-matrix interactions	Hypermethilation	35
***TIMP3***	22q12	epithelial-mesenchymal interactions	Hypermethilation	4,37
***WIF1***	12q14	Transcriptional regulator	Hypermethilation	32,52
***σ-14-3-3***	1p36	Signal transduction	Hypermethilation	30,34

**Table 3:** Deregulated miRNA in squamous cell carcinoma of the oral region (OSCC).

Cellular function	microRNAs	Expression in OSCC
*Proliferation and apoptosis*	miR-137, miR-193a, miR-133a, miR-133b, miR-503, miR-15a	Down-regulated
	miR-21, miR-24 and miR-184	Up-regulated
*Metastasis*	miR-222 and miR-138	Down-regulated
	miR-211 and miR-31	Up-regulated
*Chemoresistance*	miR-21	Down-regulated
	miR-23a, miR-214, miR-98	Up-regulated
